# Tracking in atomic detail the functional specializations in viral RecA helicases that occur during evolution

**DOI:** 10.1093/nar/gkt713

**Published:** 2013-08-10

**Authors:** Kamel El Omari, Christoph Meier, Denis Kainov, Geoff Sutton, Jonathan M. Grimes, Minna M. Poranen, Dennis H. Bamford, Roman Tuma, David I. Stuart, Erika J. Mancini

**Affiliations:** ^1^Division of Structural Biology, The Wellcome Trust Centre for Human Genetics, University of Oxford, Headington, Oxford OX3 7BN, UK, ^2^Institute for Molecular Medicine Finland (FIMM), University of Helsinki, 00290 Helsinki, Finland, ^3^Department of Environmental Research, Siauliai University, Vilniaus gatvė 88, 76285 Siauliai, Lithuania, ^4^Diamond Light Source Limited, Harwell Science and Innovation Campus, Didcot, Oxfordshire OX11 0DE, UK, ^5^Department of Biosciences, University of Helsinki, Biocenter 2, PO Box 56, 00014 Helsinki, Finland, ^6^Institute of Biotechnology, University of Helsinki, Biocenter 2, PO Box 56, 00014 Helsinki, Finland and ^7^Astbury Centre for Structural Molecular Biology and School of Cellular and Molecular Biology, University of Leeds, Leeds LS2 9JT, UK

## Abstract

Many complex viruses package their genomes into empty protein shells and bacteriophages of the *Cystoviridae* family provide some of the simplest models for this. The cystoviral hexameric NTPase, P4, uses chemical energy to translocate single-stranded RNA genomic precursors into the procapsid. We previously dissected the mechanism of RNA translocation for one such phage, ɸ12, and have now investigated three further highly divergent, cystoviral P4 NTPases (from ɸ6, ɸ8 and ɸ13). High-resolution crystal structures of the set of P4s allow a structure-based phylogenetic analysis, which reveals that these proteins form a distinct subfamily of the RecA-type ATPases. Although the proteins share a common catalytic core, they have different specificities and control mechanisms, which we map onto divergent N- and C-terminal domains. Thus, the RNA loading and tight coupling of NTPase activity with RNA translocation in ɸ8 P4 is due to a remarkable C-terminal structure, which wraps right around the outside of the molecule to insert into the central hole where RNA binds to coupled L1 and L2 loops, whereas in ɸ12 P4, a C-terminal residue, serine 282, forms a specific hydrogen bond to the N7 of purines ring to confer purine specificity for the ɸ12 enzyme.

## INTRODUCTION

Viruses protect their genome by condensing it into a compartment, the virion. Many complex viruses rely on rapid encapsidation by energy-dependent transport of the nucleic acid into an empty preformed capsid (procapsid). This process requires the presence of portal complexes, which are conduits for nucleic acid molecules, and molecular motors that convert the chemical energy gained from nucleoside triphosphate (NTP) hydrolysis into mechanical movement, resulting in nucleic acid translocation.

Some viruses, including herpesvirus and tailed double-stranded DNA (dsDNA) bacteriophages, package their genome using a multi-protein packaging motor (terminase) that transiently assembles at a single vertex (1–4). These complexes are relatively elaborate, consisting of a large dodecameric portal that is an integral part of the capsid and an oligomeric transiently associated terminase, neither of which can work in the absence of the other. The ATPase-nuclease terminase subunit is responsible for recruiting the viral DNA to the procapsid. Compacting relatively stiff dsDNA into a small volume of the procapsid has a high energy cost. Single-molecule experiments have revealed that viral packaging proteins can exert forces as high as 110 pN on dsDNA, making them some of the strongest known biological motors (5).

Similarly, dsRNA bacteriophages of the *Cystoviridae* family (bacteriophages ɸ6 through to ɸ14, and ɸ2954) encapsidate single-stranded RNA (ssRNA) genomic precursors into procapsids (6). However, their packaging machinery is less complex, consisting of a hexamer that is at the same time the physical portal and the active genome translocating motor (7,8). Although this motor shares the same function of translocating the genomic nucleic acid into the procapsid, the challenges differ between ssRNA and dsDNA. ssRNA is significantly more flexible (persistence length l_p_ ∼1–2 nm) than dsDNA (l_p_ ∼50 nm) (9), and the packaging densities are less than those found for dsDNA viruses (10); therefore, high forces are probably not required. However, naturally occurring ssRNAs, such as the genomic precursors, exhibit extensive local secondary structure (11,12), and thus the packaging motor has to exhibit helicase activity.

The lipid-enveloped bacteriophages of the *Cystoviridae* family infect Gram-negative bacteria, mainly plant-pathogenic *Pseudomonas* species (13) and share similarities with the members of the *Reoviridae* family, including bluetongue virus and rotavirus (14). Their genome of ∼14 kb consists of three dsRNA segments small (S), medium (M) and large (L), which are sequentially encapsidated as ssRNA precursors into the icosahedrally symmetric procapsid by the packaging NTPase P4 (15–23).

**Figure 1.**
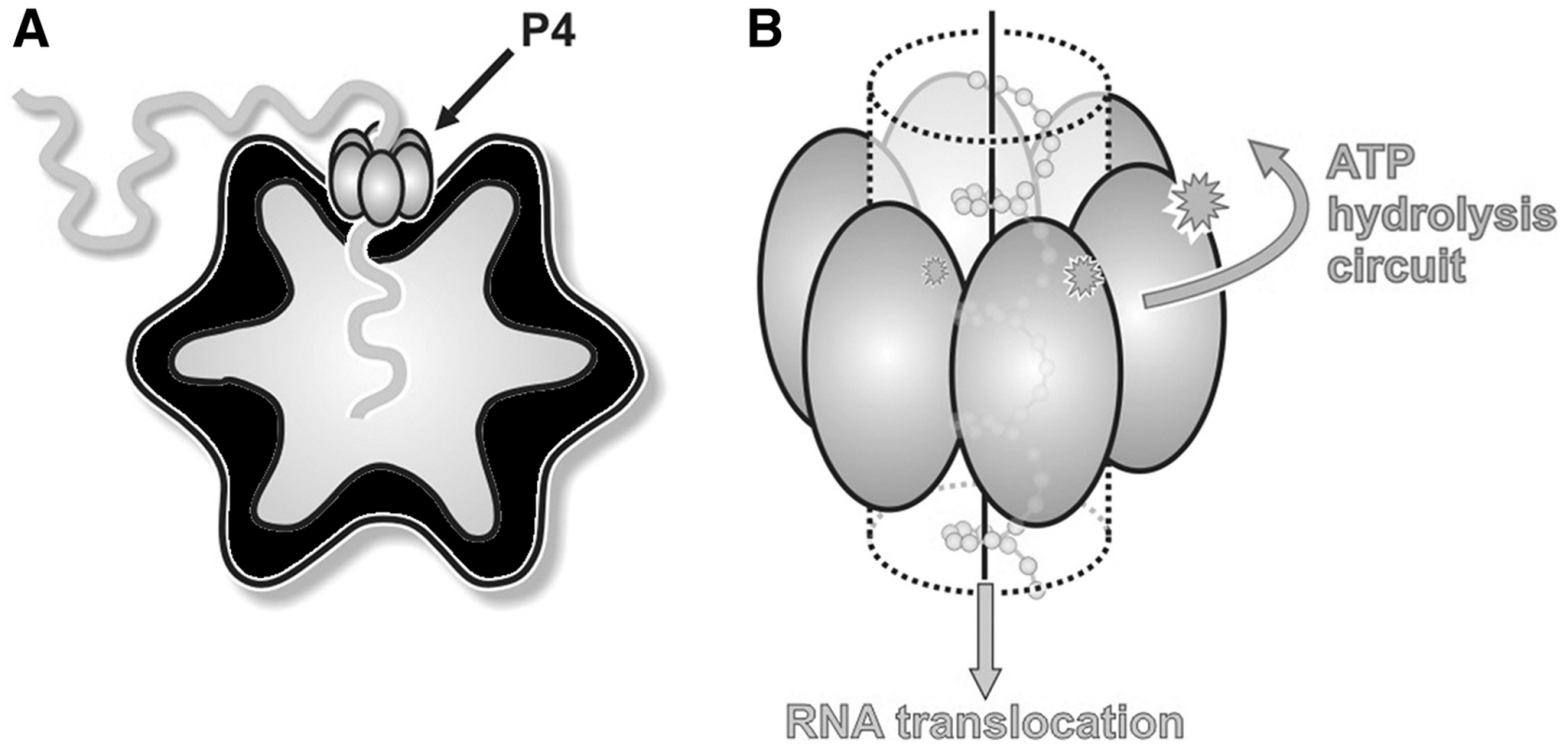
The cystovirus P4 protein, a molecular packaging motor. (**A**) Cartoon showing the position of the P4 hexamer (grey) on the empty cystovirus procapsid (black) while packaging ssRNA. (**B**) Cartoon model of the mechanism of RNA translocation by P4. The energy derived from the hydrolysis of ATP is mechanically converted to the translocation of single-stranded ssRNA.

P4 NTPases are structural components of the procapsid, built by co-assembly of 120 copies of the major structural protein P1 with ∼10 copies of the viral RNA-dependent RNA polymerase P2, 10 hexamers of P4 and 12 trimers of the assembly cofactor P7 (24) (Figure 1). In bacteriophage ɸ6, P4 hexamers nucleate procapsid assembly *in vitro* (7,25), are essential for genome packaging (21) and also have a role in transcription (21,26). Up to 12 P4 hexamers lie on the 5-fold symmetry axes of facets of the procapsid (16,24,27), creating a symmetry mismatch. Although the P4 hexamer constitutes the packaging motor, the specificity for viral RNA is mediated by RNA-binding sites on the P1 shell, which recognize three distinct packaging signals on the genomic precursors (28,29).

Previous studies have revealed the structure and mechanism of ɸ12 P4 (30–32). P4 is a protein of ∼35 kDa, which can assemble into a hexameric ring. NTP-binding sites are located on the external perimeter of the ring at the interfaces between adjacent subunits, whereas the nucleic acid binding sites are found in the central channel (31) (Figure 1). P4 proteins are the only known RNA-specific helicases belonging to helicase Superfamily 4 (SF4) (33). SF4 encompasses mainly DNA helicases and is characterized by five conserved sequence motifs (H1, H1a, H2, H3 and H4) (34). Motifs H1, H1a and H2 are involved in nucleotide binding and hydrolysis, whereas H3 is involved in the coupling of NTP hydrolysis to nucleic acid translocation, and H4 in oligonucleotide binding. Crystal structures of ɸ12 P4 at different key catalytic states of the protein unveiled a power stroke mechanism by which a conformational change associated with sequential NTP hydrolysis is responsible for RNA translocation (31,35,36).

P4 NTPases show little sequence similarity; however, they are believed to share a common architecture and mechanism of action. When recombinant P4 proteins are studied in isolation, they show variation in their *in vitro* biochemical properties (Table 1): ɸ8 and ɸ13 P4 NTPases form stable complexes with RNA and their ATPase activities are strongly stimulated by RNA (ɸ8 has no detectable ATPase activity in absence of RNA), whereas ɸ6 and ɸ12 P4s bind RNA transiently and are only weakly stimulated; the isolated P4 hexamers of ɸ8 and ɸ13 have measurable helicase activities *in vitro* in contrast to ɸ6 P4, which only acquires processive helicase activity in the context of the procapsid (30); the ɸ12 P4 hexamer has low translocation processivity and lacks helicase activity (36); the NTPase activity of ɸ12 P4 is specific to purine bases (26), whereas the other P4s can also accept pyrimidine bases (8,40). These differences in biochemical properties are presumably reflected in the hexamer architecture and structural details of different domains. To gain further insights into RNA loading, interaction and translocation mechanisms and the structural evolution of these packaging enzymes, we have solved the crystal structures of three additional P4 proteins, from ɸ8, ɸ13 and from the prototype virus of the cystoviral family, ɸ6. We also report here the structural and/or biochemical characterization of ɸ12 P4 mutants to explain nucleotide specificity and RNA recognition. We compare these structures with that of wild-type ɸ12, whose structure has already been reported (31), creating a series of structurally related viral packaging motors.

**Table 1.** Biochemical properties of wild-type and mutant P4 proteins

P4	ϕ6	ϕ8	ϕ12	ϕ13	ϕ12 S292A	ϕ12 Q278A	ϕ12 Y288A	ϕ12 ΔTTS202-204	ϕ12 TTS202-204LKK
					Nucleotide binding			RNA binding L1 loop	
Mol weight (kDa)	35	34.1	35.1	37.6	35.1	35.1	35.1	35.1	35.1
Quaternary structure and stability	Hexamer^7^ (+ATP/ADP)	Hexamer (8)	Hexamer (26)	Hexamer (8)	Hexamer	Hexamer	Hexamer	Hexamer	Hexamer
		Controlled ring opening (37)	Frequent ring opening (38)						
*K_M_*_(ATP)_ mM	0.19 ± 0.03 (8)	NA	1.50 ± 0.04 (36)	0.40 ± 0.05 (8)		2.20 ± 0.5			
*k_cat_ s^-1^*	0.19 ± 0.06 (8)	0.00 ± 0.05 (39)	0.84 ± 0.12 (36)	1.60 ± 0.05 (8)	0	0.25 ± 0.13	0	0	0
*K_M_*_(ATP_) mM with 1 mM polyC	ND	0.17 ± 0.01 (39)	0.49 ± 0.02 (36)	ND		2.10 ± 0.1			
*k_cat_ s^-1^* with 1 mM polyC	ND^7^	6.40 ± 0.20 (39)	2.52 ± 0.07 (36)	ND	0	0.96 ± 0.05	0	0	0
NTP specificity	All (8)	All (8)	Purine base (26)	All (8)					
ssRNA binding	Kd > 1 Mm (8)	Kd<1 µM (8,39)	Kd> 1 mM (26,36)	Kd<1 µM (8)					
ssRNA translocation	Weak (7)	Strong	Weak (36)	Strong (8)					
		Processive (36,39)							
COD (helicase) activity	Only in PC (8)	Strong (8)	None (8)	Weak (8)					
RNA binding site	ND	L1 (LKK) (37)	L2 (K241) (30)	ND					

## MATERIALS AND METHODS

### Cloning, expression and purification

Recombinant full-length P4 from ɸ8, ɸ13 and C-terminally truncated ɸ8 P4Δ281 (missing residues 281–321) and ɸ6 P4Δ310 (missing residues 310–331) were expressed from plasmids pSJ1b (41), pDK3 (8), pDK10 (42) and pJTJ7.3/7 (43), respectively. Point mutations were introduced into ɸ12 *gene 4* using plasmid pPG27 (32) as a template to introduce amino acid substitutions S252Q, R272A, Q278A, S292A, Y288A and TTS202-204 by site-directed mutagenesis (QuikChange, Stratagene) following the manufacturer recommendations. The corresponding plasmids were designated as pDK33, pDK35, pDK30, pDK31, pDK29 and pDK249 respectively. The insertion of LKK instead of TTS (residues 202–204) was introduced by amplifying the N-terminal portion of the P4 gene with primers 1 and 2 (Supplementary Table S1) and the C-terminus part with primers 3 and 4. PCR products were digested with NdeI/AflII (N-terminal part) and AflII/EcoRI (C-terminal part) and ligated into pT7-7 vector at NdeI-EcoRI sites. Sequencing was used to confirm the mutations.

Recombinant P4 proteins were expressed in *Escherichia coli* BL21(DE3) or B834(DE3) and purified to homogeneity as previously described (31,32,42). Briefly, *E. coli* cells were grown at 37°C in Luria-Bertani medium until OD_540nm_ reached 0.5–0.6. Cultures were then chilled on ice and induced with 1 mM isopropyl-β-thiogalactopyranoside. Induced cells were further incubated for 12–14 h at 17–18°C, harvested by centrifugation and lysed with a French pressure cell. P4 proteins were purified by chromatography: Heparin and Q-sepharose columns (GE Healthcare) followed by size exclusion chromatography (Superdex 200, GE Healthcare).

Cloning, expression, purification and characterization of C-terminally His-tagged ɸ8 P4 (ɸ8 P4His), which exhibits full RNA-induced ATPase activity, was described previously (44).

### Crystallization

Crystallization conditions of the P4 proteins have been previously described (32,42). In brief, crystals of ɸ6 P4Δ310 proteins were grown at 24°C from a 3.5 mg/ml protein solution in 20 mM HEPES (pH 8.0), 5 mM MgCl_2_, 2 mM CaCl_2_, 5 mM adenosine diphosphate (ADP) and 100 mM NaCl, and they appeared after 9 months in drops in which 3 µl of protein had been mixed with 3 µl of a reservoir solution consisting of 6% PEG 4000 and 90 mM sodium acetate (pH 4.5). Crystals were cryo-protected by transferring them into reservoir solution with a final glycerol concentration of 25% before freezing in a nitrogen-gas stream at −173°C.

From a 12 mg/ml protein solution, ɸ13 P4 crystals were grown at 20°C using 100 mM Tris–HCl (pH 7.0), 900 mM trisodium citrate and 200 mM NaCl as precipitant. Crystals were cryo-protected as ɸ6 P4Δ310, but using a final glycerol concentration of 20%.

The ɸ8 P4 crystals were grown at 24°C in 100 mM sodium acetate (pH 4.6) and 2.2 M ammonium sulphate as a precipitant. Drops consisted of 0.9 µl of protein at a concentration of 3 mg/ml, 0.9 µl of reservoir solution and 0.4 µl of 100 mM dithiothreitol (DTT). Crystals of ɸ8 P4Δ281 obtained from a protein solution concentrated to 5 mg/ml appeared in 100 mM Tris (pH 8.0) and 18% PEG 1000. Crystals were cryo-protected following the protocol for ɸ6 P4Δ310.

Crystals of ɸ12 P4 mutants were obtained in a solution composed of 10% PEG 1500 in 100 mM sodium acetate (pH 4.8) and 5 mM AMPcPP. Crystals of wild-type ɸ12 P4 with UTP were obtained with the same precipitant and 5 mM UTP.

### Data collection and structure determination

Data collection was performed as previously detailed (32,42), and all data were indexed, integrated and scaled using HKL2000 (45). Crystallographic statistics for the data are detailed in Supplementary Table S2.

Structures of ɸ12 P4 mutants ɸ12 P4-Q278A and ɸ12 P4-S292A were solved by molecular replacement using the program PHASER (46) with wild-type ɸ12 P4 (PDB code 1W4B) as the search model.

The structure of ɸ13 P4 was solved by single-wavelength anomalous dispersion as described elsewhere (47). The substructure was determined using the program SHELX (48), and phases were refined using SHARP (49). After 6-fold non-crystallography symmetry averaging using General Averaging Program (unpublished program available from D. I. Stuart or J. M. Grimes), an interpretable electron density map was obtained into which the structure could be built.

The structure of ɸ6 P4 was solved by molecular replacement with the crystal structure of the ɸ13 P4 as a search model. The search model included one hexamer in which each chain was truncated to the conserved ATPase core of the protein. A weak molecular replacement solution comprising two truncated hexamers was found by the program AMoRe (50). The preliminary phases were greatly improved by 12-fold non-crystallographic symmetry averaging and phase extension from low resolution using General Averaging Program. The last 34 residues of the ɸ6 P4Δ310 construct were not visible in the electron density; their absence might be due to proteolysis, which would explain the long crystallisation period.

The structure of ɸ8 P4 was initially solved by single-wavelength anomalous dispersion from crystals of the selenomethione labelled protein in space group *P*622 containing one monomer in the asymmetric unit. HKL2MAP (48) was used to identify the selenium sites, which were then fed into PHENIX AUTOSOL (51), resulting in an interpretable electron density map for the ATPase core domain. The electron density corresponding to the rest of the protein was not interpretable owing to the statistically disordered crystal reported previously (42). The hexameric P4 was formed by applying the crystallographic symmetry and used as search model for molecular replacement with the program PHASER (46) to find a solution for ɸ8 P4 (*R*32 space group) and ɸ8 P4Δ281 (*P*2_1_2_1_2 space group).

Manual building was performed with the program COOT (52) and restrained refinement (with TLS) with either AUTOBUSTER (53) or REFMAC5 (54). The final models were validated with MolProbity (55). Refinement statistics are provided in Supplementary Table S2; in summary; the resolution (Å)/R-factor(%)/R-free(%) for the structures were ɸ6 P4: 2.8/21.7/24.4, ɸ8 P4-His: 3.1/29.6/30.9, ɸ12 P4 UTP: 1.9/19.4/20.4, ɸ13 P4: 1.7/16.4/18.8.

### Hydrogen-deuterium exchange mapping

Previously published hydrogen-deuterium exchange (HDX) data for ɸ8 P4 were used (37) and mapped onto the high-resolution structure presented in this work using average rate colouring as described (37).

### ATPase activity of mutants

ATPase activity of ɸ12 P4-binding site mutants was assayed using the EnzChek phosphate assay kit (Invitrogen) (39).

### Evolutionary analysis of structures

The coordinates of the ATPase core of P4 from ɸ8 (residues 104–261) were submitted to the DALI Server (56), a program that identifies and ranks proteins by structural similarity. The DALI search returned 47 proteins, which have significant structural similarity to P4. All these proteins were then truncated to their core ATPase domains, and using the program SHP superimposed onto one another, and a matrix of structural relationships was calculated (57).

## RESULTS AND DISCUSSION

### Overall fold

**Figure 2.**
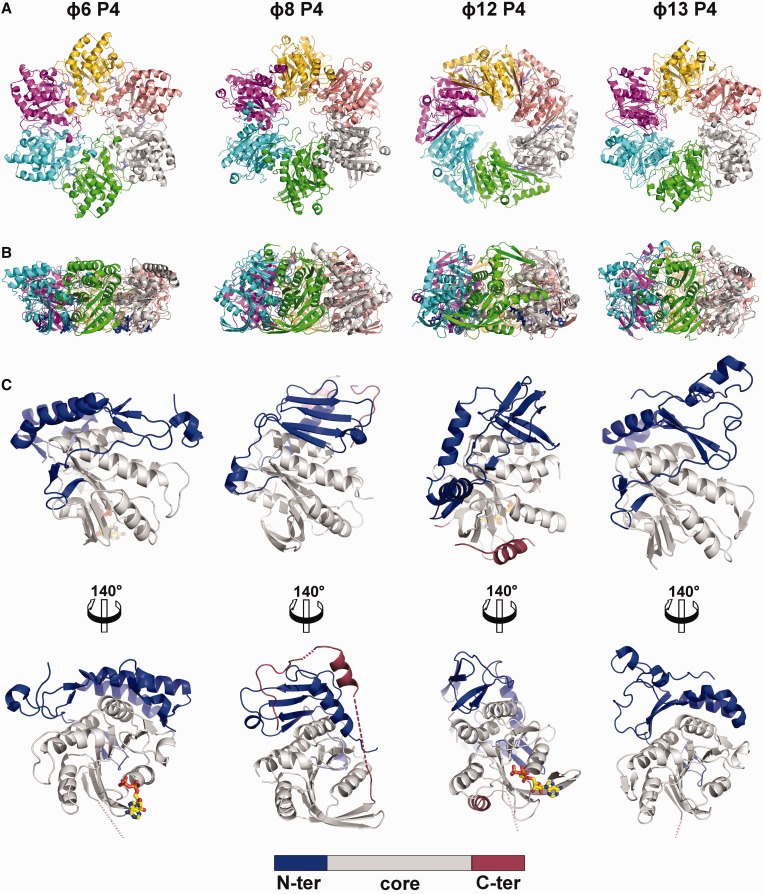
The overall fold of cystoviral P4 proteins. (**A**) The P4 hexamers of bacteriophages ɸ6, ɸ8, ɸ12 and ɸ13 (left to right) are viewed from the top and coloured by chain. (**B**) Side view of the P4 hexamers. (**C**) The panel shows structures of monomeric P4 in two orientations, the upper orientation of the monomer corresponding to the one depicted in cyan in (B); the lower one has undergone a rotation of 140°C to show the C-terminal domains. The core domain is coloured in grey, the N-terminal domain in blue and the C-terminal domain in red. Nucleotides, if present, are depicted as sticks with carbon, oxygen, nitrogen and phosphorus atoms coloured in yellow, red, blue and orange, respectively. Dotted lines represent the disordered region of the proteins.

All P4 proteins form a hexameric ring with a central channel varying in size from 13 to 21 Å (30 Å for ɸ8 P4Δ281) and external diameter of ∼100 Å (Figure 2). However, the hexamers have different charge distributions on their surfaces (Supplementary Figure S1) and different outline shapes: ɸ6 P4, ɸ8 P4 and ɸ13 P4 form hexagonal notched rings, whereas ɸ12 P4 has a smoother contour. The subunit interface within hexamers varies in size from ∼1500 to 1900 Å^2^, and the number of hydrogen bonds, salt bridges and hydrophobic interactions shows substantial variation (Supplementary Table S3). The interfaces within the P4 hexamers are more polar than expected for a stable oligomer. This is because rings of hexameric helicases are generally required to open to load the nucleic acid strand into the central cavity (Table 1) (58,59). The rounder ɸ12 P4 subunits bury the biggest surface area and form the highest number of hydrogen bonds and salt bridges, whereas the interaction area is least for ɸ8 P4, which harbours fewer hydrogen bonds and only three salt bridges. The buried area does not correlate with P4 ring stability. For example, ɸ12 P4 has been shown to exhibit frequent ring opening unless it is bound to the procapsid (38), leading to low translocation processivity (36). On the other hand, ɸ8 P4 is a processive translocase and opens only during loading a new RNA strand into the central channel (37). Ring stability correlates instead with the fraction of buried polar interactions (hydrogen bonds and salt bridges) per buried area. The less stable ɸ6 and ɸ12 hexamers have 0.016 and 0.018 polar contacts per Å^2^ respectively, whereas the more stable ɸ8 and ɸ13 exhibit values of 0.13 and 0.15, respectively.

### ATPase core domain

**Figure 3.**
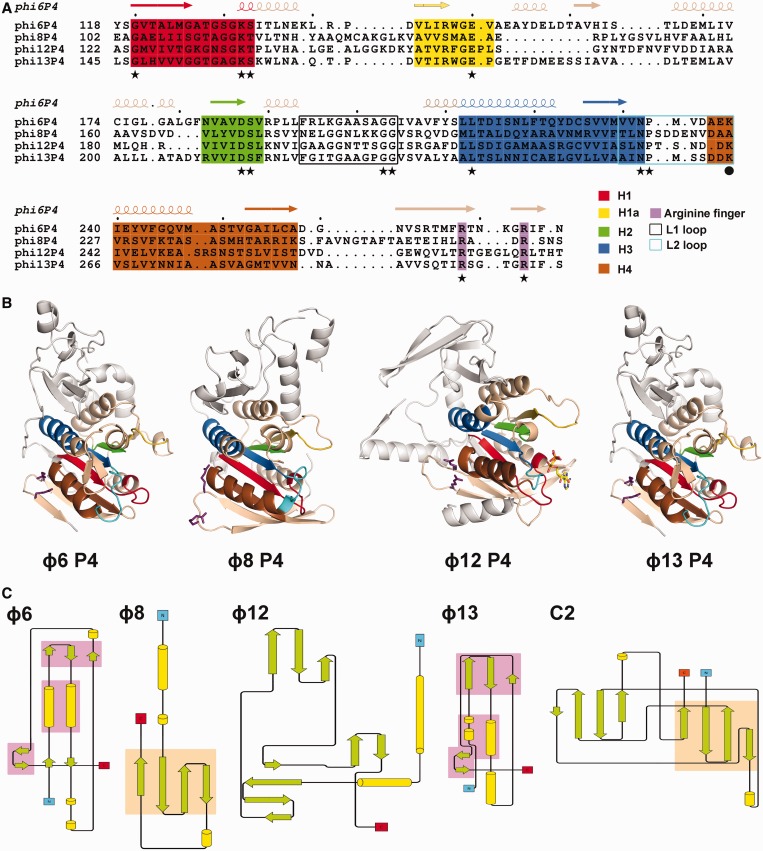
Structural conservation between P4 proteins. (**A** and **B**) Sequence and structural conservation of the helicase motifs in P4 proteins. Motifs H1, H1a and H2 are involved in nucleotide binding and hydrolysis, H3 is involved in the coupling of NTP hydrolysis to nucleic acid translocation, and H4 in oligonucleotide binding. Motifs H1, H1a, H2, H3, H4 are coloured in red, yellow, green, blue and brown, respectively; the arginine fingers are coloured purple, whereas the L1 and L2 loops are black and cyan, respectively. (A) Structure-based acid sequence alignment of the ATPase core domain of ɸ6, ɸ8, ɸ12 and ɸ13 P4. Functionally important residues that are conserved amongst the different cystoviruses are indicated by stars, whereas a sphere marks the lysine in loop L2 (K241 in ɸ12 P4), which is not conserved in ɸ8 P4. (B) Cartoon representations of ɸ6, ɸ8, ɸ12 and ɸ13 P4 structures in equivalent orientations. The arginine fingers and the nucleotides are shown in a ball-and-stick representation. The colour coding is the same as in (A). (**C**) Topology diagrams of the N-terminal domains of ɸ6, ɸ8, ɸ12 and ɸ13 P4. Secondary structural elements are coloured in green (strands) and yellow (helices). Topologically similar domains are shaded in pink (ϕ6 and ϕ8) and orange (ϕ8 and C2). The topology for C2 was derived from PDB entry 2ENP.

Within the hexamer, the different P4 monomers adopt similar orientations and can be divided into three domains: an N-terminal region (110–150 residues), a central core NTPase domain of ∼160 residues and a smaller C-terminal domain (∼40–50 residues) (blue, grey and red, respectively, in Figure 2). Strikingly, despite low overall sequence conservation ranging from 9 to 21% amino acid sequence identity, the key structural features of the ATPase core domain (motifs H1, H1a, H2, H3 and H4) are well-conserved (Figure 3A andB). The ATPase domain is a Rossmann-type nucleotide-binding domain consisting of a twisted seven-stranded β-sheet with mixed parallel and antiparallel topology flanked by five helices. Residues previously demonstrated to be critically important in the mechano-chemically coupling of ATP hydrolysis to RNA translocation in ɸ12 P4 (35) are structurally conserved in other P4s (Figure 3A andB, Table 2), except for one residue in motif H4 (residue K241 in ɸ12 P4), which has no equivalent in ɸ8 P4 (see explanation for this later in the text). It is therefore likely that all cystoviral P4 NTPases use an RNA translocation mechanism similar to that described for ɸ12 P4 (31), although details may vary, especially for ɸ8 P4 where a tight coupling between ATPase activity and RNA binding is observed (Table 1).

**Table 2.** Conserved residues and their function within φ6, φ8, φ12 and φ13 P4 proteins

Amino acid	φ6	φ8	φ12	φ13	Function	Walker motif
Lysine	K132	K116	K136	K159	Phosphate binding	H1
Serine/Threonine	S133	T117	T137	S160	Phosphate binding	H1
Glutamate	E150	E141	E160	E176	Catalytic base	H1a
Aspartate	D187	D171	D189	D213	Coordinate Mg	H2
Asparagine	N232	N216	N234	N258	Sensor motif	H3
Lysine	K239	K185	K241	K265	RNA binding	H4
Serine	S250	S237	S252	S277	Sensor motif II	H4
Arginine	R268	R263	R272	R294	Arginine finger	
Glutamine			Q278		Base stacking	
Arginine	R273	R266	R279	R299	Arginine finger	
Tyrosine/Phenylalanine	F275	F247	Y288	F301	Base stacking	

**Figure 4.**
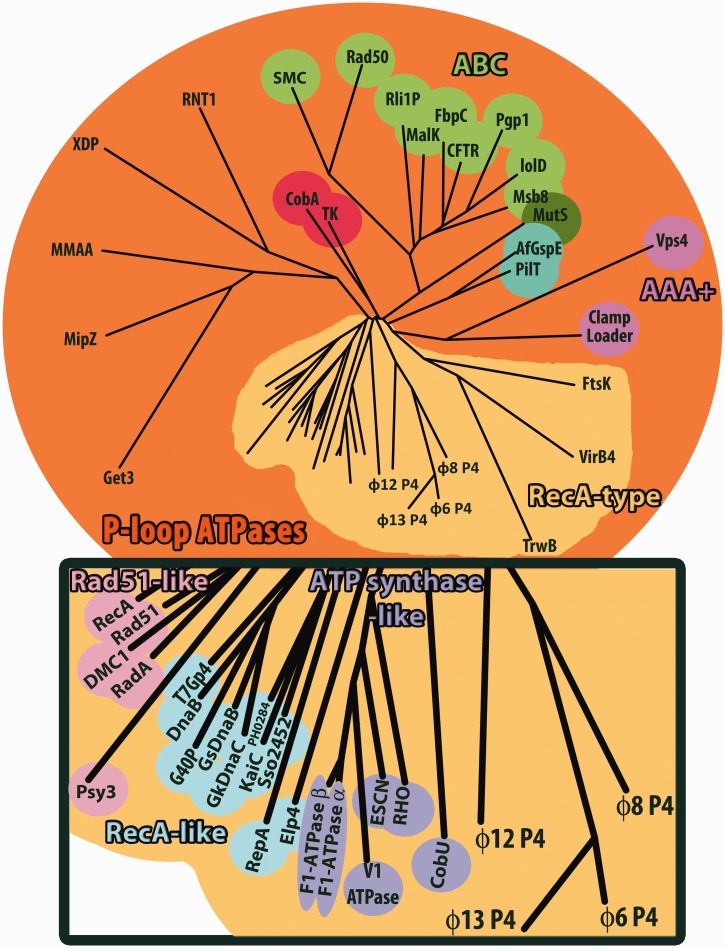
Structure-based phylogenetic tree of ATPase enzymes. The matrix of evolutionary distances was calculated with SHP (56). The rectangle corresponds to a close-up view of the members of the RecA family. Abbreviations (In alphabetical order; Protein Data Bank accession codes are quoted in brackets): AfGspE, archaeal secretion ATPase, (2Oap); CFTR, Cystic Fibrosis Transmembrane Conductance Regulation, (1Xmi); Clamp Loader, eukaryotic clamp loader, (1Sxj); CobA, corrinoid adenosyltransferase, (1G64); CobU, adenosylcobinamide kinase/adenosylcobinamide phosphate guanylyltransferase, (1Cbu); DMC1, meiotic recombination protein, (2Zjb); DnaB, *Thermus aquaticus* DNAb, (2Q6t); Elp4, elongator complex protein 4, (4A8j); ESCN, prototypical T3ss ATPase EscN, (2Obl); F1-ATP Synthase-α, ATP synthase subunit-α heart isoform, (2Jj1); F1-ATPase-β, bovine mitochondrial F1-ATPase, (1E1r); FbpC, Fe(3+) ions import ATP-binding protein FbpC, (3Fvq); FtsK, DNA translocase FtsK, (2Iut); G40P, ATPase domain of G40P, (3Bh0); Get3, ATPase Get3, (3Sja); GkDnaC, *Geobacillus kaustophilus* DnaC, (2Vyf); GsDnaB, *Geobacillus stearothermophilus* DnaB, (2R6c); IoID, *Aquifex Aeolicus* ABC transporter, (2Pcj); KaiC, Circadian clock protein kinase KaiC, (3K0e); MalK, maltose/maltodextrin import ATP-binding protein, (2Awn); MipZ, bacterial cell division regulator protein MipZ, (2Xit); MMAA, methylmalonic aciduria type A protein, (2Www); Msb8, *Thermotoga maritima* Abc transporter ATPp-binding protein, (1Vpl); MutS, DNA mismatch repair protein MutS, (1Ewq); P-gp, multidrug resistance protein Pgp-1, (4F4c); PH0284, Upf0273 Protein Ph0284, (2Dr3); PilT, twitching motility protein PilT, (2Gsz); Psy3, Platinum sensitivity protein 3, (4Dt1); Rad50, Dna Double-Strand Break Repair Rad50 Atpase, (3Qku); Rad51, DNA repair protein Rad51, (1Szp); RadA, DNA repair and recombination protein RadA, (4Dc9); RecA, Recombinase A, (1Mo4); RepA, regulatory protein RepA, (1G8y); Rho, transcription termination factor Rho, (3Ice); Rli1p, translation initiation factor, (3J16); RNT1, regulator of nonsense transcripts 1, (2Wjy); SMC, chromosome partition protein, (4I99); Sso2452, putative uncharacterized protein, (2W0m); T7Gp4, T7 DNA Primase/Helicase, (1Cr1); TK, thymidine kinase, (2Ja1); TrwB, conjugal transfer protein TrwB, (1E9r); V1-ATPase, V-Type sodium ATPase, (3VR4); VirB4, type IV secretory pathway Virb4 components-like protein, (4Ag6); Vps4, vacuolar protein sorting-associated protein 4, (3Eih); XDP, Xpd/Rad3 related DNA helicase, (3Crv).

Structural classification based on the ATPase core domain shows that cystovirus P4 proteins are closely related to each other and only distantly related to other P-loop ATPases (Figure 4 and Supplementary Figure S2). They most closely resemble RecA-type ATPases (35), such as ATP synthase-like proteins (RHO, F1-ATPase, etc.), RecA-like proteins (RepA, T7 gp4, etc.) and Rad51-like protein (Rad51, RecA, etc*.*). Many of these proteins are involved in nucleotide repair and recombination and have similar functional properties to P4 proteins. This indicates that the cystoviral P4 proteins form a distinct subfamily of RecA-type ATPases.

### N-terminal domain

The structural conservation across P4 proteins of the central ATPase core domains does not extend to the N- and C-terminal domains. Most of the N-terminal domain residues of P4 from ɸ6 and ɸ8 are visible in our crystal structures (starting from amino acid residues 2 and 12, respectively), whereas ɸ13 P4 lacks the first 32 residues [which are predicted to be disordered (60)]. In all P4 structures, the N-terminal domain covers the apical part of the hexamer (Figure 2), and in ɸ12 P4, an N-terminal domain α-helix projects from one subunit to the adjacent one, giving the hexamer a more rounded appearance. ɸ6 P4 lacks such a helix and might stabilize the hexamer by strengthening subunit interfaces with nucleotides. ɸ6 P4 is the only P4 that needs nucleotides and divalent cations to form hexamers (7). It is also conceivable that NTP binding triggers a conformational change in the ɸ6 P4 subunits allowing them to form hexamers. Interestingly, ɸ8 and ɸ13 P4s also lack such a stabilizing helix; however, the first 12 and 31 residues, respectively, are not visible in the crystal structures and might play such a stabilizing role.

The N-terminal domains of cystoviral P4s are highly divergent (Figures 2, 3B and C). However in ɸ6 and ɸ13, more than half of their residues can be superimposed with a root-mean-square deviation of 2.1 Å, including two parallel helices and two small anti-parallel β-sheets, creating a topologically identical sub-domain (Figure 3C). In ɸ8 and ɸ12, the N-terminal domains have higher secondary structure content but are completely unrelated to each other and to those in ɸ6 and ɸ13. In ɸ12 P4, the N-terminal domain is composed of two orthogonal α-helices and three anti-parallel β-sheets (Figure 3C). The ɸ8 P4 N-terminal domain is composed of two helices separated by a four-stranded antiparallel β-sheet (Figure 3C). Structural alignment searches against the PDB database returned no significant matches for any of the N-terminal domains, aside from a weak structural similarity (43 of 87 residues within 3.7 Å) of ɸ8 P4 to one half of a C2 domain (domain involved in targeting proteins to cell membranes; Figure 3C). Intriguingly, ɸ8 lacks the P8 nucleocapsid protein layer present in other cystoviruses so that P4 proteins (together with P1 shell) interact directly with the viral lipid membrane (10).

### C-terminal domain

The C-terminal domain of P4 comprises ∼40–50 amino acid residues downstream of the ATPase core (Figure 2) expected to be located at the bottom of the hexamer and to be essential for binding to the capsid protein P1 (38,61). The C-terminal domains of P4 proteins diverge substantially. In ɸ6 and ɸ13, the C-termini are predicted to be disordered with little secondary structure (60), and indeed, no density for these domains could be found in our crystal structures. In contrast, the corresponding regions in ɸ8 and ɸ12 are predicted to be mostly ordered (60) with a C-terminal helix preceded by a flexible loop. In P4 ɸ12, the strand following the arginine finger motifs extends back into the ATP-binding site contributing two residues (Y288 and S292), which help position the nucleotide ring (see later in the text). The density for the amino acid chain then disappears to re-emerge into a C-terminal helix stacked at the bottom of the hexamer (Figure 2). In P4 ɸ8, the strand following the arginine fingers motifs does not extend as far as the ATP-binding site but instead climbs back along the side of the hexamer (partially disordered) to re-emerge into as C-terminal helix at the top of the hexamer (Figure 2B), followed by a loop that dives into the central channel restricting its diameter by more than half (see later in the text for more discussion on the C-terminal domain).

### Nucleotide binding site

**Figure 5.**
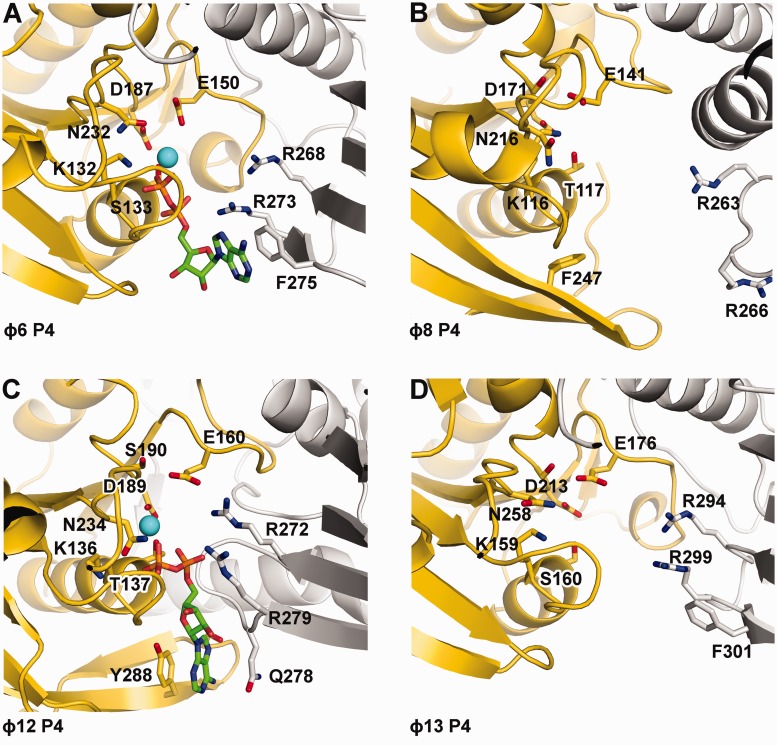
Cartoon representation of the nucleotide binding sites of ɸ6 (**A**), ɸ8 (**B**), ɸ12 (**C**) and ɸ13 (**D**) P4s. Within hexamers, adjacent monomers are coloured in yellow and grey. Nucleotides (ADP), if present, are depicted as sticks with carbon atoms coloured in green. Oxygen, nitrogen and phosphorus atoms are coloured in red, blue and orange, respectively, and the position of Mg^2+^ (ɸ12 P4) or Ca^2+^ (ɸ6 P4) is indicated with a cyan sphere.

The ɸ6 P4 was crystallised with ADP-Mg^2+^ bound in the nucleotide binding site, whereas P4 from ɸ8 and ɸ13 were crystallized in their apo form. As for ɸ12 P4, and other hexameric NTPases, the nucleotide binding sites in ɸ6 P4 are located at the interfaces between neighbouring subunits. The ADP phosphate groups are bound via the conserved Walker A (H1) motif residues (K132, S133) (Figure 5); a conserved glutamate E150 (H1a) is positioned to catalyse the nucleophilic attack on the γ-phosphate, whereas D187, a conserved aspartate in the Walker B motif (H2), co-ordinates the magnesium ion. A sensor motif detecting the presence or absence of the γ-phosphate of NTP and modulating allosteric transitions of the RNA binding loop L2 in response to ATP binding and hydrolysis was identified in P4 from ɸ12 (N234) (31). The equivalent residue in ɸ6 P4, N232, is positioned to contact the γ-phosphate of the NTP (Figure 5) and might fulfil the same role. As the mechanism of NTP binding and hydrolysis is similar, it is likely that the equivalent conserved residues in P4 from ɸ8 and ɸ13 (Figure 5 and Table 2) play analogous roles.

It has been shown that ɸ12 P4 possesses two essential ‘arginine fingers’ (35). We find that all P4 proteins follow this unusual pattern (Figure 5 and Table 2). Arginine fingers can contact the γ-phosphate of the triphosphate from a neighbouring subunit, and the insertion of this residue in a catalytic site is believed to stabilize the transition state, thus facilitating ATP hydrolysis. Arginine fingers in P4 proteins are all contributed from the same region (a loop between two strands in the C-terminal region) but display different conformations (Figure 5). In P4 from ɸ6, ɸ12 and ɸ13, the arginine fingers are pointing towards the catalytic sites, making the subunits competent and primed for hydrolysis. However, in ɸ8 P4, these residues are displaced >8 Å from that position and therefore cannot contribute to catalysis. This suggests that in ɸ8 P4, extensive conformational changes occur as a consequence of nucleotide and/or oligonucleotide binding, which render the enzyme competent for catalysis. Indeed, nucleotide binding kinetics revealed a first-order rate limiting step, which is consistent with a conformational change associated with ATP binding (39,62).

In RecA-like ATPases, bound nucleotides are stabilized by stacking of the adenine moiety between side chains, but these side chains are not conserved and are contributed from different regions. In RepA and T7 helicases, the ATP base stacks against residues belonging to the subunit carrying the catalytic site. In ɸ12 P4 (31), as in RepA (63), the nucleotide base is sandwiched between Y288 from the catalytic subunit and Q278 from the neighbouring subunit. In ɸ6 P4, a much looser stacking of the nucleotide base is observed, with only one side chain (F275) stabilizing the adenine ring (Figure 5). From our structures, we predict similar loose arrangements in P4 from ɸ8 and ɸ13 where F247 (from the same subunit) and F301 (from a neighbouring subunit) seem to be in the correct orientation to stack the nucleotide base. The difference in the arrangement of the nucleotide binding motifs is likely to explain the mechanism of base-specific hydrolysis in different P4s. Of the P4s, only ɸ12 is purine specific, with pyrimidines also being accepted by ɸ16, ɸ18 and ɸ13 (Table 1).

**Figure 6.**
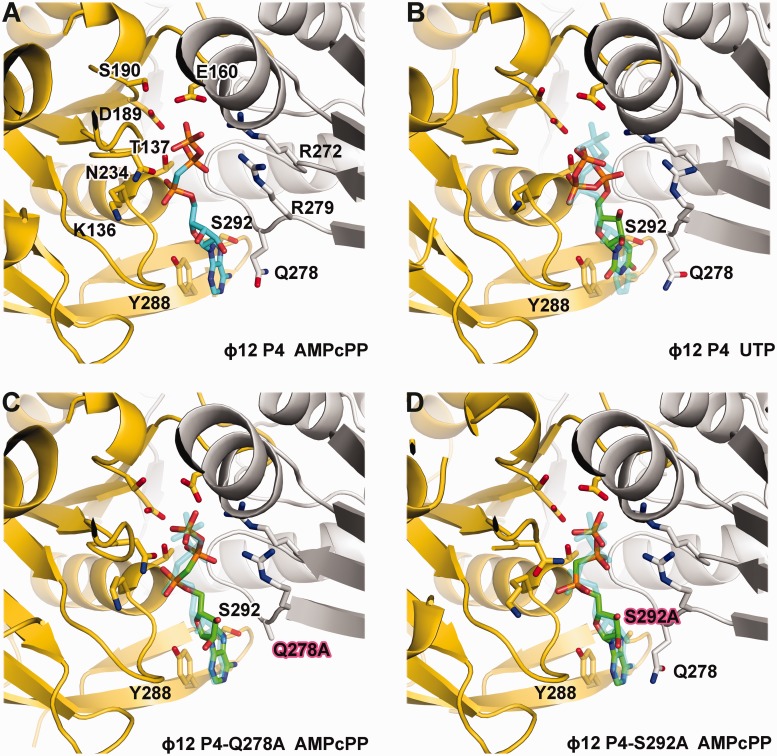
Cartoon representation of the nucleotide binding site of ɸ12 P4. (**A**) Wild-type ɸ12 P4 bound to non-hydrolysable ATP analogue AMPcPP (PDB: 1W48) or (**B**) to UTP. (**C**) Q278A mutant bound to AMPcPP. (D) S292A mutant bound to AMPcPP. Within hexamers, adjacent monomers are coloured in yellow and grey. AMPcPP bound to wild-type ɸ12 P4 is depicted in sticks, and the carbon atoms are coloured cyan (A), whereas carbon atoms in the UTP bound to ɸ12 P4 (B) and AMPcPP bound to the P4 mutants Q278A and S292A (C and **D**) are coloured in green. Oxygen, nitrogen and phosphorus atoms are coloured in red, blue and orange, respectively. (B–D) The position of the AMPcPP bound to wild-type P4 is represented in transparent for comparison.

To understand this catalytic mechanism in detail, we performed side-directed mutagenesis of the residues in ɸ12 P4 involved in binding the nucleotide ring and analysed the mutants structurally and biochemically. In ɸ12 P4, the stacking interaction is critical for nucleotide binding, as replacement of the tyrosine with alanine (Y288A) completely abolished ATP binding and ATPase activity (Table 1) so that the apoprotein structure is found even in the presence of ATP (data not shown). However, the mutation Q278A had only a moderate effect on ATPase activity and virtually no effect on the structure of the bound ATP analogue AMPcPP when compared with the wild-type (Figure 6A and C), primarily increasing the K_M_ as a result of reduced nucleotide affinity (Table 1). Hence, the stacking interactions primarily determine nucleotide affinity but not specificity. A specific feature in ɸ12 P4 is a hydrogen bond between the hydroxyl of S292 and N7 of the purine ring. The substitution S292A did not prevent ATP binding but completely abolished ATPase activity owing to misplacement of the triphosphate moiety in the active site (Figure 6D). A displacement is also seen when the AMPcPP bound wild-type structure is compared with that of UTP bound hexamer (Figure 6A and B). This confirms that pyrimidine triphosphates can bind the hexamer without being hydrolysed (36) and should act as competitive inhibitors. Indeed, we find that UTP effectively competes with ATP and inhibits hydrolysis (data not shown). Hence, purine specificity is achieved by locking the base by hydrogen bonding to the N7 site of a purine. The correct coordination of the base results in the precise alignment of the nucleotide that is essential for catalysis so that UTP is misaligned and not hydrolysed. This is probably the mechanism underpinning the dependence of helicase efficiency on the type of nucleotide. For example, T7 gp4 helicase activity is optimal in presence of dTTP (58).

### Nucleic acid binding site

It has been proposed that P4 hexamers bind nucleic acid through their central channel via two protruding loops named L1 and L2 (31) (Figure 3A andB, Supplementary Figure S3). Mutagenesis studies confirmed that these loops are essential for nucleic acid binding and translocation (30,35,37). Structurally homologous loops were reported to bind ssDNA and ssRNA, respectively, in crystals of the E1 helicase of bovine papilloma virus and Rho of *E. coli* (59). The L1 loops in P4 are rich in residues that contribute to flexibility (in ɸ12 P4 they are disordered), whereas the L2 loops are mainly composed of hydrophilic residues, amongst them a lysine, which in ɸ12 P4 (K241) was shown to be essential for RNA binding (35). The structures of P4 from ɸ6 and ɸ13 show ordered L1 loops, which line the central channel and contact the L2 loops (Supplementary Figure S2). The L2 loops are found with lysine residues (K239 and K265, respectively) projecting towards the centre of the channel, in the same position as K241 in ɸ12, suggesting a conserved mechanism for binding and translocating RNA. Although the L2 loop of ɸ8 P4 contains hydrophilic residues (DDENVD), it does not project a lysine side chain towards the central channel. Nevertheless, the L1 loop contains a motif (LKK) that has been shown to be crucial for RNA binding (35). The first lysine of this motif (K185) is found in the equivalent position to K241 of ɸ12 P4 and is also seen interacting with D220 of loop L2. We therefore postulate that K185 (loop L1) in ɸ8 P4 plays the same role in RNA binding as K241 (loop L2) in ɸ12 P4, and that the coupling of the movement of the L1 and L2 loops to ATP hydrolysis via motion of helix 6, as proposed for ɸ12, may be a general feature of all P4 molecules (Supplementary Figure S2). The importance of the L1 loop is further supported by mutational analysis in ɸ12 P4: deleting L1 loop central residues T202-T203-S204 or mutating them into the equivalent residues of ɸ8 P4 (LKK) completely abolishes the ATPase activity (Table 1). This demonstrates that the integrity of the L1 loop is essential for ATP hydrolysis, despite being distal to the ATP active site.

### RNA loading in ɸ8 P4 and the structural basis of processive translocation

The ɸ8 P4 ATPase activity is tightly coupled to ssRNA translocation, as it will only hydrolyse ATP in the presence of ssRNA. As noted earlier in the text, the RNA binding motif LKK in loop L1 is located in the middle of the central channel (37). Nucleic acids are likely to bind in the channel, ensuring topological enclosure of the strand and processive translocation.

**Figure 7.**
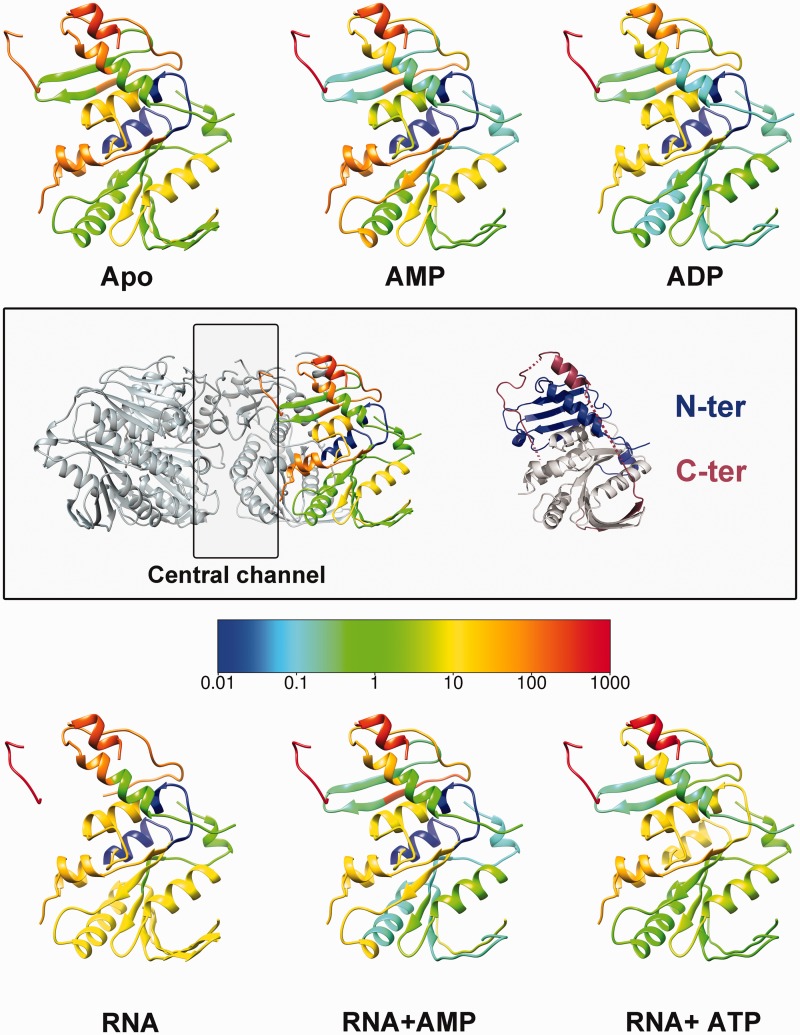
Mapping of HDX data on the ɸ8 P4 structure. HDX rates are coloured from slow-exchange (blue) to fast-exchange rates (red). Previously measured HDX rates (53) for ɸ8 P4 in the presence/absence of AMP, ADP, ATP and RNA (as indicated) were mapped onto the ɸ8 P4 monomer structure. The central box shows on the left, the orientation of all the monomers of the figure within the hexamer, and on the right, the same monomer in which the N- and C-terminal domains are coloured in blue and red, respectively.

**Figure 8.**
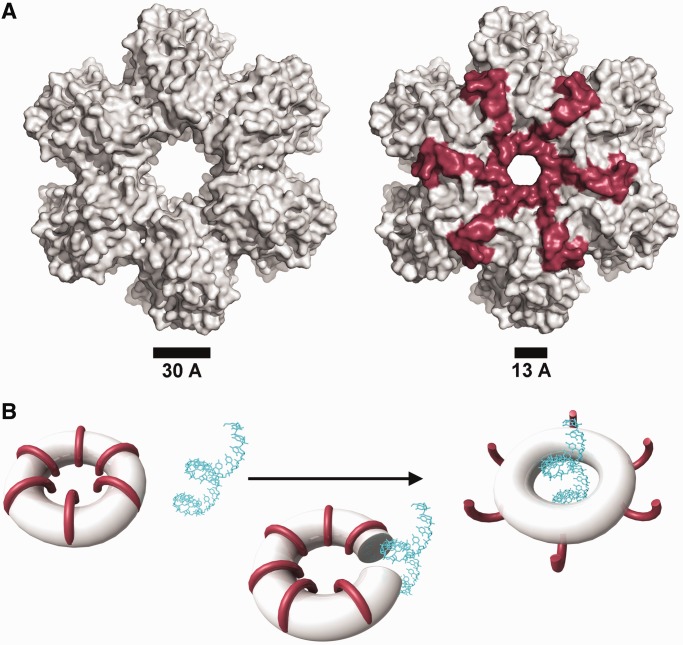
The C-terminal domain of ɸ8 P4. (**A**) Surface presentation of the ɸ8 P4Δ281 (left) and the full-length protein (right). The C-terminal domain is coloured in red. (**B**) A model for ssRNA induced displacement of the C-terminal domain in ɸ8 P4 hexamer.

Based on transient cooperative exposure of subunit interfaces to HDX on RNA binding (residues 198–209 in Figure 7), it was suggested that RNA enters the central channel via a transient ring opening (37). The deletion of the C-terminal portion of the protein (residues 282–321) more than doubles the diameter of the central channel (from 13 to 30 Å), as the C-terminus wraps upwards from the base of the hexamer, along the inter-subunit cleft, to stick down into the central channel (Figure 8). As the C-terminal domain is (i) necessary for ATP hydrolysis (data not shown), (ii) restricts the diameter of the central channel and (iii) blocks the interface through which RNA is thought to be loaded, we postulate that the C-terminal region needs to be displaced by RNA for ring opening and subsequent ATP hydrolysis to occur. To verify this hypothesis, previous HDX experiments (37) were further analysed by mapped to the ɸ8 P4 structure.

The C-terminal region exhibits the fastest HDX within the protein (Figure 7). However, the distal C-terminal portion that extends into the central channel is marginally protected in the absence of RNA and becomes fully exposed only on addition of RNA, implying that this region becomes further exposed presumably by expulsion from the central channel (Figure 8B). Thus, it appears that ɸ8 P4 has developed a specific mechanism to regulate ATPase activity and couple it with ssRNA binding such that RNA displaces the C-terminal domain, to allow ATP hydrolysis to occur. This would explain the tight coupling observed between ATP hydrolysis and translocation.

## CONCLUSION

The current study broadens our understanding of the mechanism used by dsRNA bacterial viruses to package RNA genome during assembly. Interestingly, P4 proteins are only remotely related to packaging ATPases of dsDNA viruses such as gp17 from bacteriophage T4 (64) or pUL15 from Herpex Simplex virus 1 (65), which have more complicated portal complexes. Recently, however, it has been suggested that the ATPase of the phi29 DNA packaging motor is a member of the hexameric AAA+ superfamily (66), indicating that the mechanism of nucleic acid packaging might be similar.

A structure-based phylogeny (Figure 4) suggests that the RecA-like proteins may be the closest cellular relatives of the P4, with ɸ12 being the most similar to the cellular proteins, ɸ8 being rather divergent and ɸ6 and ɸ13 rather similar to each other and intermediate in terms of divergence from the cellular proteins. These structural variations map onto the various functional specializations of the molecules so that although the motors have a common catalytic mechanism, they have developed somewhat different specificity and control mechanisms. We identify a specific hydrogen bond (serine 292 and N7 of the purine ring) responsible for the purine specificity of ɸ12 P4 catalysed NTP hydrolysis reaction and find that an extraordinary insertion of the C-terminal peptide into the central channel of the hexamer explains the tight coupling of ATPase activity and RNA translocation in ɸ8. Furthermore, the ɸ8 P4 structure revealed a novel mechanism of power transduction to the RNA in which RNA is engaged with the L1 loop, which, in turn, is coupled to the L2 loop. Comparison between the P4 structures suggest that coupling between the two loops may be a general mechanistic feature of P4 and perhaps other SF4 helicases. Overall, the P4 machine represents a remarkable test bed where, by virtue of high mutational rates over long periods of time, nature has been able to devise a range of functional variations on the basic theme of regulated RNA translocation, resulting in an array of systems where although the molecular engine remains largely similar, the ignition and transmission systems have diverged markedly.

## ACCESSION NUMBERS

Coordinates and structure factors of ADP-bound ɸ6 P4Δ310, ɸ8 P4, ɸ8 P4Δ281, UTP-bound ɸ12 P4, AMPcPP-bound ɸ12 P4-Q278A and AMPcPP-bound ɸ12 P4-S292A and ɸ13 P4 have been deposited in the Protein Data Bank under accession codes 4BLO, 4BWY, 4BLQ, 4BLR, 4BLS, 4BLT and 4BLP, respectively.

## SUPPLEMENTARY DATA

Supplementary Data are available at NAR Online.

## FUNDING

UK Medical Research Council (MRC); Academy of Finland [255342 and 256518 to D.H.B. as well as 250113 and 256069 to M.M.P.]; Sigrid Juselius Foundation (to DH.B. and M.M.P.); European Union Structural Funds programme [VP1-3.1 -ŠMM-07-K-03-069 to D.K.]; Academy of Finland Centre of Excellence in Virus Research 2006–2011 (to R.T.); The Wellcome Trust [075491/Z/04]; a Royal Society University Research Fellow (to E.J.M.); a Junior Research Fellow at Oriel College, Oxford (to K.E.O.). Funding for open access charge: Medical Research Council, UK.

*Conflict of interest statement*. None declared.

## Supplementary Material

Supplementary Data
