# Supplementary material for: Tracking in atomic detail the functional specializations in viral RecA helicases that occur during evolution
Source: Nucleic Acids Res. 2013 Aug 10;41(20):9396–410. doi: 10.1093/nar/gkt713 (PMC3814363; doi:10.1093/nar/gkt713)
Supplement: Supplementary Data [file supp_41_20_9396__index.html]

Tracking in atomic detail the functional specializations in viral RecA helicases that occur during evolution — Tracking in atomic detail the functional specializations in viral RecA helicases that occur during evolution — Supplementary Data 

# Tracking in atomic detail the functional specializations in viral RecA helicases that occur during evolution

## Supplementary Data

files

**Files in this Data Supplement:**

- Supplementary Data - pdf file
