# Supplementary material for: Tracking in atomic detail the functional specializations in viral RecA helicases that occur during evolution
Source: Nucleic Acids Res. 2013 Aug 10;41(20):9396–410. doi: 10.1093/nar/gkt713 (PMC3814363; doi:10.1093/nar/gkt713)
Supplement: Supplementary Data [file supp_gkt713_nar-01566-h-2013-File010.pdf]

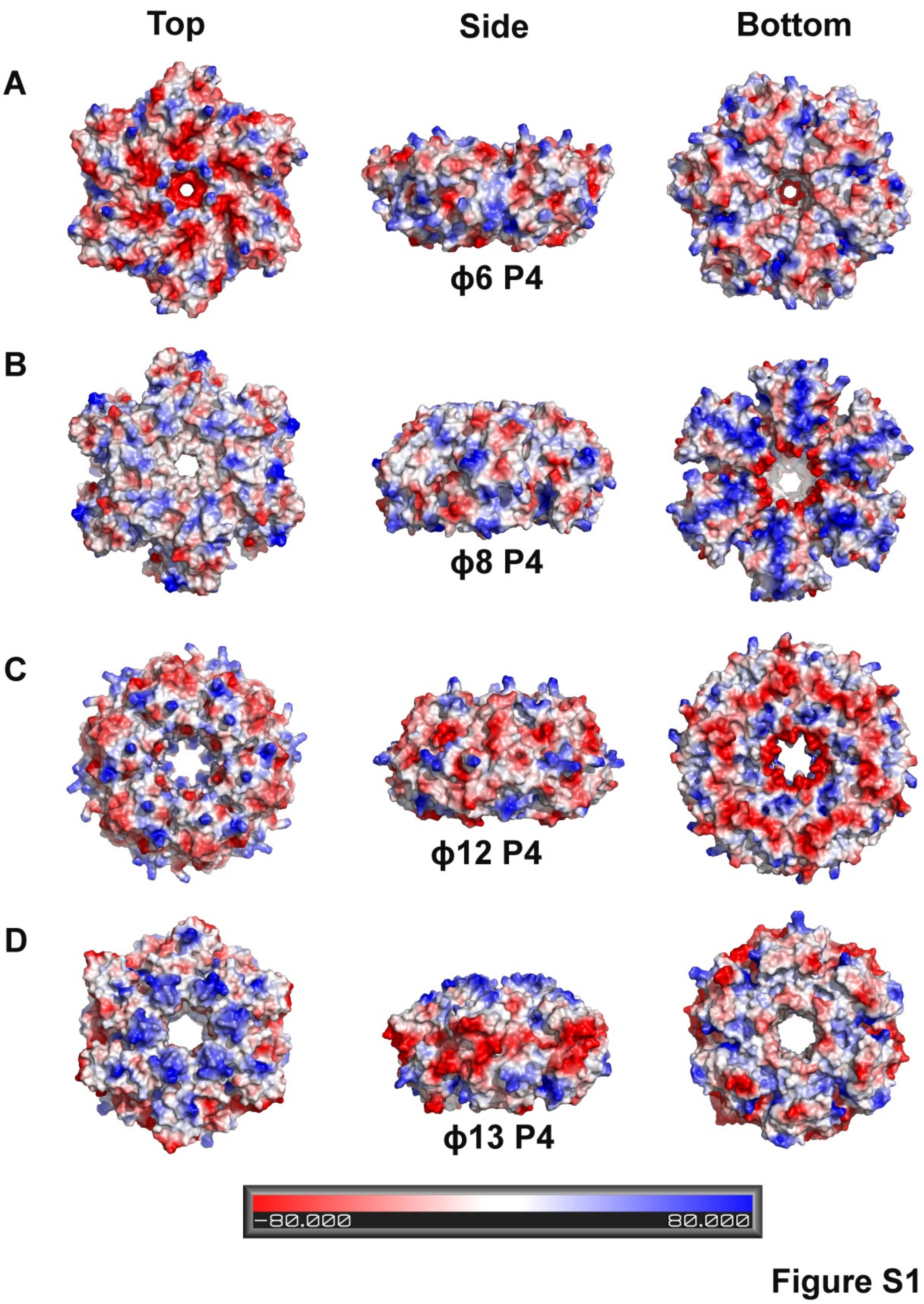

Supplementary figure S1: Overall electrostatic charge distribution on hexameric P4 proteins: ϕ6 (A), ϕ8 (B) and ϕ12 (C) and ϕ13 (D). From left to right are represented the top, side and bottom view.

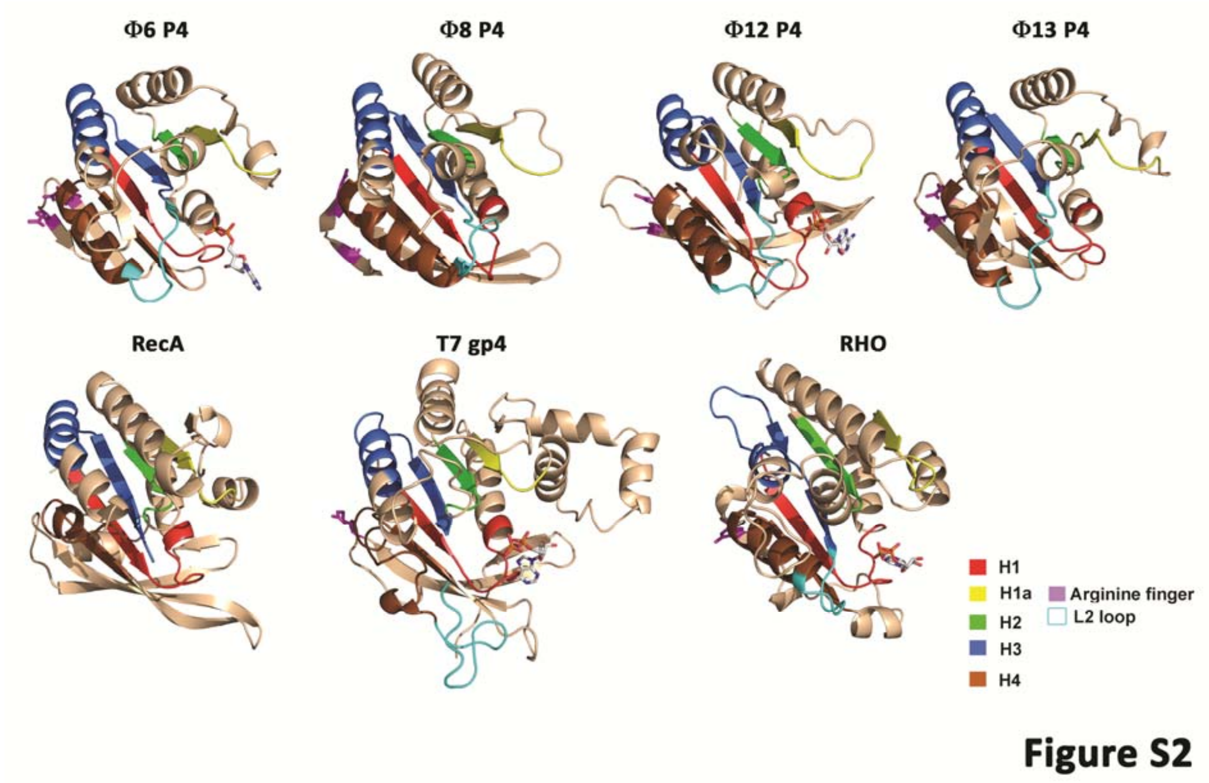

Supplementary figure S2: Structural superimposition of the core ATPase domain of P4 proteins and structurally related proteins: *E.Coli* RecA (RecA) (PDB 2REB), T7 gp4 helicase (T7gp4) (PDB 1CR1), and *E.Coli* Rho helicase (RHO) (PDB 3ICE). The colour scheme for conserved motifs (H1-H4) is the same used in sequence alignment in Figure 3.

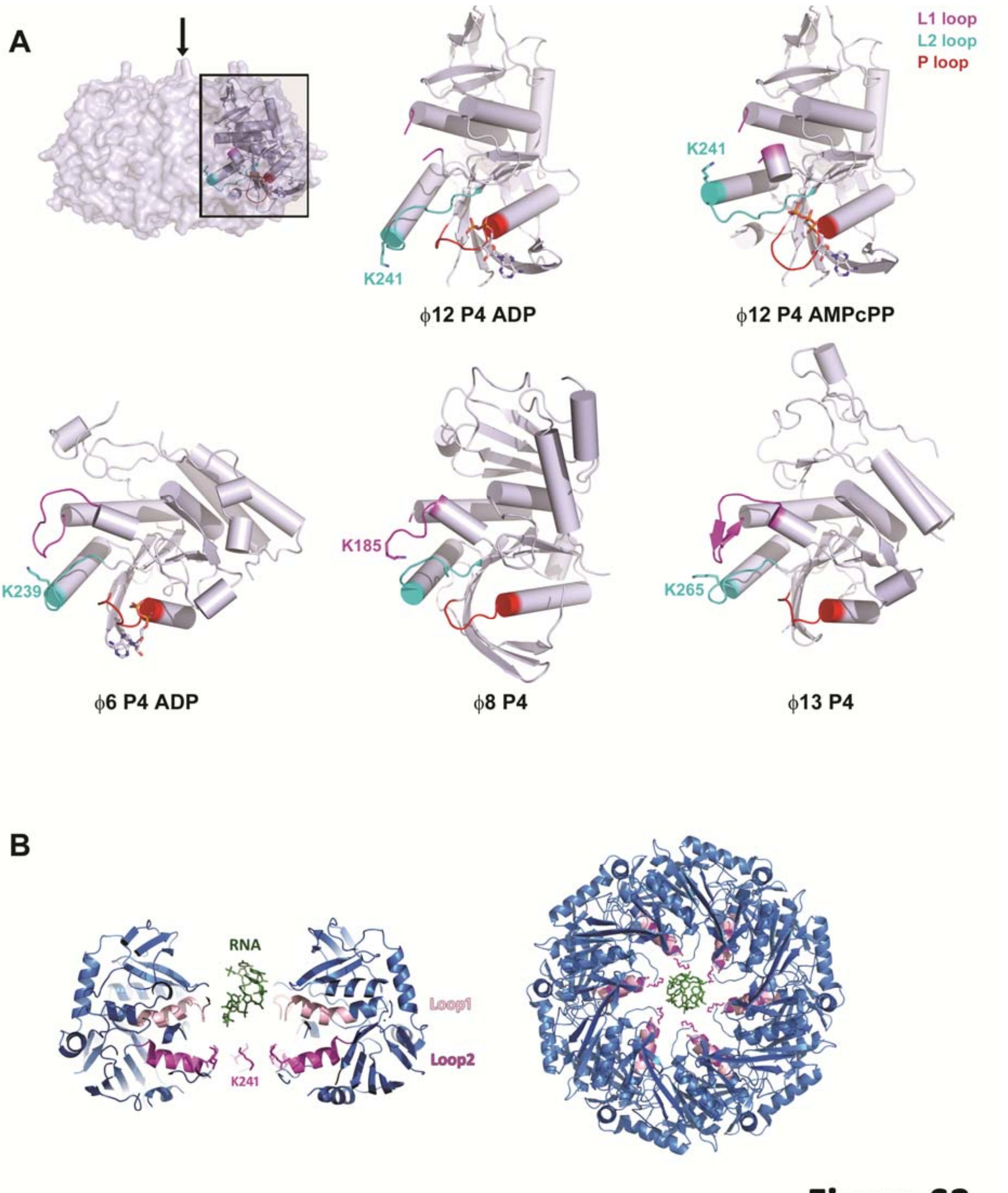

Figure S3

Supplementary figure S3: (A) The central L1 and L2 loops. Structures of monomeric P4 proteins are drawn in the same orientation. The lysines involved in RNA binding are depicted in sticks. The L1, L2 and P-loop are coloured in pink, cyan and red respectively. (B): Model for RNA binding. Plausible model for RNA binding within the hexamer based on fitting of the "staircase" RNA structure from *E.Coli* Rho (PDB 3ICE) into the central channel of the Φ12 P4 hexamer. Left, side view with only two subunits shown for clarity and loops L1, L2 highlighted in light pink and magenta. Right, top view showing the position of the RNA in the P4 hexamer.

Mutation	Mutagenic oligonucleotide	Plasmid
**TTS202-204LKK**	[1]5’- GGAGTGA<u>CATATG</u>ATCCATCTGTACGA - 3’<br>[2]5’- CGTAAC<u>CTTAAG</u>GGTATTTCTCGT - 3’<br>[3]5’- CTCGG<u>CTTAAG</u>ATTACCACCAGC - 3’<br>[4]5’- CTC<u>GAATTC</u>TCAGTTGGAGGTGAGA - 3’	pDK27
**ΔTTS202-204**	5’- GCAGCTGGTGGTAATGGTGGTATTTCTCGTGGC - 3’<br>5’- GCCACGAGAAATACCACCATTACCACCAGCTGC - 3’	pDK49
**S252Q**	5’- CAAGGAAGCGAGCCGC**CAG**AACAGCACGTCTCTC - 3’<br>5’- GAGAGACGTGCTGTT**CTG**GCGGCTCGCTTCCTTG - 3’	pDK33
**R272A**	5’- GCAAGTACTGACA**GCT**ACCGGTGAAGGTCTGC - 3’<br>5’- GCAGACCTTCACCGGT**AGC**TGTCAGTACTTGC - 3’	pDK35
**Q278A**	5’- CGGTGAAGGTCTG**GCA**CGCCTGACACACACTC - 3’<br>5’- GAGTGTGTGTCAGGCG**TGC**CAGACCTTCACCG - 3’	pDK30
**Y288A**	5’- CACACACTCTGCAGACGAGT**GCC**GGCGAGCATTCGGTCCTCACC - 3’<br>5’- GGTGAGGACCGAATGCTCGCC**GGC**ACTCGTCTGCAGAGTGTGTG - 3’	pDK29
**S292A**	5’- GTTATGGCGAGCAT**GCG**GTCCTCACCATCCAC - 3’<br>5’- GTGGATGGTGAGGAC**CGC**ATGCTCGCCATAAC - 3’	pDK31

Supplementary table S1. Primers used for mutagenesis.

Data collection	ϕ6 P4	ϕ8 P4	ϕ8 P4His	ϕ8 P4Δ281	ϕ12 P4 UTP	ϕ12 P4-Q278A	ϕ12 P4-S292A	ϕ13 P4
Beamline	SRS 14.2	ESRF ID29	DLS IO2	ESRF ID14-EH4	SRS 14.2	ESRF ID14-EH4	ESRF ID14-EH4	ESRF ID14-EH2
Space group	P1	P622	R32	P21212	I222	I222	I222	P212121
Unit cell (Å, º)	a=93.8, b=95.3, c=98.3, α=82.5, β=86.2, γ=64.8	a=b=100.7, c=65.7	a=b=197.7, c=562.6	a=138.9, b=159.6, c=100.3	a=105.4, b=129.3, c=159.1	a=105.6, b=132.3, c=159.9	a=104.4, b=131.7, c=159.3	a=97.2, b=116.2, c=149.8
Wavelength (Å)	0.8701	0.9771	0.9798	1.0056	0.9755	0.9756	0.9759	0.933
Resolution (Å)	20.0-2.8 (2.90-2.80)	50.0-2.0 (2.07-2.00)	50.0-3.1 (3.21-3.10)	72.5-2.8 (2.85-2.78)	20.0-1.9 (1.97-1.90)	20.0-2.6 (2.69-2.60)	50.0-2.4 (2.49-2.40)	50.0-1.7 (1.76-1.70)
Completeness (%)	97.0 (88.2)	99.8 (100)	100 (99.8)	99.9 (99.9)	100.0 (99.8)	94.6 (93.4)	93.1 (70.7)	99.3 (99.5)
Redundancy	3.0 (2.6)	39.4 (32)	12.7 (11.1)	11.6 (11.9)	7.2 (5.8)	6.6 (4.8)	9.6 (6.2)	4.3 (4.3)
I/σ(I)	8.3 (1.5)	101.8 (20.8)	8.6 (1.3)	14.6 (3.3)	29.4 (4.9)	7.1 (7.1)	17.6 (4.2)	19.5 (2.4)
Rmerge (%)	18.6 (85.9)	9.7 (37.3)	0.31 (100)	12.7 (88.5)	7.7 (38.1)	15.1 (28.5)	10.6 (29.2)	7.0 (65.5)
**Refinement**								
Resolution (Å)	19.9/2.8		45.0-3.1	50.0-2.8	20.0-1.9	50.0-2.6	20.0-2.4	19.8-1.7
Rwork/Rfree (%)	21.7/24.4		29.6/30.9	21.4/22.9	19.4/20.4	25.6/27.4	26.3/28.9	16.4/18.8
Rmsd bond (Å)	0.009		0.008	0.008	0.004	0.005	0.004	0.012
Rmsd angle (º)	1.05		1.05	1.01	1.01	1.02	0.94	1.1
Mean B-factor/Wilson plot (Å²)	57/51		67/68	58/68	26/18	43/46	39/31	22/17
Ramachandran plot (%) favored/allowed/outliers	97.7/100/0		94/100/1	97.8/100/0	99.5/100/0	97.9/100/0	98.7/100/0	98.7/100/0

Supplementary table S2. Data collection and refinement statistics.

	ϕ6 P4	ϕ8 P4	ϕ12 P4	ϕ13 P4
**Subunit interface (Å^2)**	1826	1605	1938	1530
**Number of Hydrogen bonds**	24	19	26	16
**Number of Salt bridges**	6	3	8	7
**Number of residues with a Buried area >30%**	73	68	84	70

Supplementary table S3. Subunit interface interactions determined by PISA (41).
